# Improving emergency department transfer for patients arriving by ambulance: A retrospective observational study

**DOI:** 10.1111/1742-6723.13407

**Published:** 2019-12-23

**Authors:** Julia Crilly, Amy NB Johnston, Marianne Wallis, John O'Dwyer, Joshua Byrnes, Paul Scuffham, Ping Zhang, Emma Bosley, Wendy Chaboyer, David Green

**Affiliations:** ^1^ Department of Emergency Medicine Gold Coast University Hospital, Gold Coast Health Gold Coast Queensland Australia; ^2^ Menzies Health Institute Queensland Griffith University Gold Coast Queensland Australia; ^3^ Department of Emergency Medicine Princess Alexandra Hospital Woolloongabba Queensland Australia; ^4^ School of Nursing, Midwifery and Social Work The University of Queensland Woolloongabba Queensland Australia; ^5^ School of Nursing, Midwifery and Paramedicine University of the Sunshine Coast Maroochydore DC Queensland Australia; ^6^ Australian eHealth Research Centre Herston Queensland Australia; ^7^ School of Medicine Griffith University Nathan Campus Brisbane Queensland Australia; ^8^ Office of the Commissioner, Queensland Ambulance Service, Department of Health Brisbane Queensland Australia

**Keywords:** advanced practice nurse, ambulance, emergency department, evaluation, patient outcomes

## Abstract

**Objective:**

Extended delays in the transfer of patients from ambulance to ED can compromise patient flow. The present study aimed to describe the relationship between the use of an Emergency Department Ambulance Off‐Load Nurse (EDAOLN) role, ED processes of care and cost effectiveness.

**Methods:**

This was a retrospective observational study over three periods of before (T1), during (T2) and after (T3) the introduction of the EDAOLN role in 2012. Ambulance, ED and cost data were linked and used for analysis. Processes of care measures analysed included: time to be seen by a doctor from ED arrival (primary outcome), ambulance‐ED offload compliance, proportion of patients seen within recommended triage timeframe, ED length of stay (LoS), proportion of patients transferred, admitted or discharged from the ED within 4 h and cost effectiveness.

**Results:**

A total of 6045 people made 7010 presentations to the ED by ambulance over the study period. Several measures improved significantly between T1 and T2 including offload compliance (T1: 58%; T2: 63%), time to be seen (T1: 31 min; T2: 28 min), ED LoS (T1: 335 min; T2: 306 min), ED LoS <4 h (T1: 31%; T2: 33%). Some measures carried over into T3, albeit to a lesser extent. Post‐hoc analyses showed that outcomes improved most for less urgent patients. The annualised net cost of the EDAOLN (if funded from additional resources) of $130 721 could result in an annualised reduction of approximately 3912 h in waiting time to be seen by a doctor.

**Conclusion:**

With the EDAOLN role in place, slight outcome improvements in several key ambulance and ED efficiency criteria were noted. During times of ED crowding, the EDAOLN role may be one cost‐effective strategy to consider.

**Key findings**

- In this single site study, modest improvements in several process measures (ambulance offload compliance, time to be seen, ED length of stay) were noted when a dedicated ambulance offload nurse role was operational.
- If funded from additional resources, the net cost of the ambulance offload nurse role could result in a reduction of patient waiting times.
- The ambulance offload nurse role is one strategy to consider, alongside other hospital strategies, to support immediate care requirements for patients arriving to ED by ambulance.

## Background

Around one in four presentations to Australian public EDs are via ambulance.^1^ The number of these ambulance arriving presentations has increased from 1.5 to 1.9 million between 2010–2011 and 2016–2017 (out of 6.2 and 7.8 million total ED presentations, respectively).^1^, ^2^ Clearly efficient processing of patients arriving by ambulance is required to ensure efficient use of resources.

From a patient‐centred and health service point of view, it is important that safe, quality care be delivered to all patients presenting to the ED, including those arriving by ambulance. This care includes having the right personnel undertaking the right processes in an environment and system that facilitates timely access to care to achieve optimal outcomes.^3^ But, delivering safe, quality care can be difficult, particularly during times of ED and hospital crowding. Delays in care can compromise patient safety and can start at the point of ED entry with patient off‐stretcher time (POST) delays (also referred to as ambulance ramping, ambulance offload delay and ambulance turnaround delays).^4^, ^5^, ^6^, ^7^, ^8^, ^9^, ^10^, ^11^, ^12^, ^13^ POST delays occur when paramedics are unable to complete transfer of clinical care of their patient to the hospital ED within a clinically appropriate timeframe due to unavailable appropriate clinical space in the ED.^14^ An ‘appropriate timeframe’ in this situation has been referred to as <15 min^4^, ^15^ or <30 min.^7^, ^8^, ^12^, ^13^, ^14^

During POST delays, not only is there difficulty accessing ED care, but ambulances are also unavailable to respond back to the surrounding community.^7^, ^9^, ^16^ While a number of recommendations around the input phase of the patient's journey (that includes patients arriving to ED by ambulance) have been made,^10^, ^17^, ^18^, ^19^, ^20^, ^21^, ^22^ increasing volumes of patients and patient flow challenges in EDs suggest further strategies are required to support health system performance. In response to unprecedented access block and ED crowding, an ED Ambulance Off‐Load Nurse (EDAOLN) role was trialled on a 24/7 basis for a 39‐day period in one Australian ED. The role of the EDAOLN included rapid triage and assessment, and commencement of initial meaningful treatment (such as initiating or arranging X‐rays, pathology and analgesia) as required. The role was not an additional funded staff member; rather it was derived from a redistribution of existing staffing arrangements. Appendix S1 provides further details of the role. The aim of the present study was to describe the relationship between the use of an EDAOLN role, ED processes of care and cost effectiveness, with comparison for urgent *versus* less urgent cases. We hypothesised that the EDAOLN role would be associated with improvements in processes of care.

## Methods

### *Study design and setting*

This was a retrospective observational study informed by a health service and nursing role evaluation framework.^23^, ^24^ The study was undertaken in a large regional public, teaching hospital in Australia, serving a population of around 515 000.^25^ The ED treated paediatrics and adults and had 67 484 ED presentations in 2012. Approximately 37% of ED presentations made to the study site arrived by ambulance.^13^

### *Sample*

**Figure 1:**
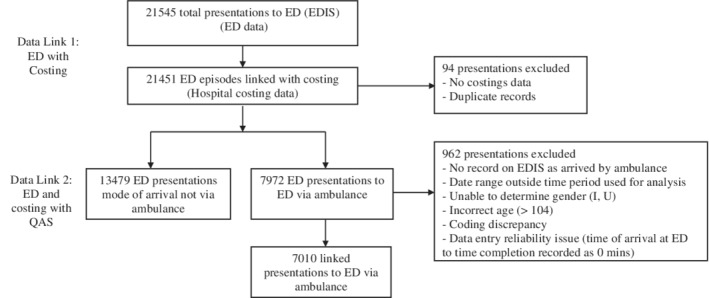
Data linkage process for sample inclusion.

The sample was drawn from all patient presentations made to the ED by ambulance, before, during and after withdrawal of the role, with each time block approximately 5 weeks: Time 1 (T1, pre‐EDAOLN) 9 July–16 August 2012, Time 2 (T2, during‐EDAOLN) 17 August–24 September 2012, and Time 3 (T3, post‐EDAOLN) 25 September–30 October 2012. The duration of the EDAOLN role (i.e. T2) was pragmatic and not based on a formal sample size calculation. Figure 1 displays the sample inclusion process.

### *Data collection*

Routinely collected data from the Queensland Ambulance Service (QAS) electronic Ambulance Report Form (eARF), the Emergency Department Information System (EDIS) and the associated hospital costing database – Transitions II database (TII) were linked and used for analysis (Appendix S2). Processes of care measures included: time to be seen by a doctor from arrival (primary measure of interest), POST, ambulance at ED turnaround time, ambulance‐ED offload compliance (i.e. <30 min), time to be seen, proportion of patients seen within the recommended Australasian Triage Scale (ATS)^26^ timeframe, ED length of stay (LoS), proportion of patients transferred, admitted or discharged from the ED within 4 h and admission rate (see Appendix S3 for definitions). Analysis also included a health economic component to establish the cost effectiveness of the EDAOLN to the ED and ambulance service.

### *Statistical analysis*

The unit of analysis was the ED presentation to capture the true extent of ED workload derived from all presentations to ED by ambulance. Descriptive statistics, presented as mean (standard deviation, SD) and median (interquartile range, IQR), were used to summarise the demographic characteristics, clinical characteristics and processes of care measures. Inferential statistics were used to identify differences between groups in T1, T2 and T3. One‐way analysis of variance (anova) and post‐hoc *t*‐tests were used for normally distributed data; Kruskal–Wallis one‐way anova (k samples; used for comparison of demographic data from patient presentations) and post‐hoc Mann–Whitney *U*‐tests were used for non‐normally distributed data (e.g. costs and post‐hoc comparison of median ED LoS). χ^2^ tests were used to compare categorical variables (e.g. sex, ATS category, diagnosis code, shift [grouped as morning, evening, night] and ED LoS <4 h – the National Emergency Access Target (NEAT) compliance measure).^27^ Sub‐group analyses were undertaken to compare profiles and processes of care measures based on (i) time of arrival (pre‐, during or post‐EDAOLN) and (ii) triage category (1/2 *vs* 3/4/5).

Costs were analysed from the perspective of Queensland Health (responsible for both hospital and ambulance services) to identify the costs and potential cost‐savings from the EDAOLN. All costs are reported in 2012 Australian dollars. A detailed cost‐effective analysis is described in Appendix S4. Despite this being a redistribution of existing staffing, as information on the impact on other patients was not captured, the economic analysis was conducted on the basis of employing an additional staff member, thereby taking a conservative approach. Furthermore, the primary cost offset identified was derived from paramedic waiting time in the ED. The cost offset associated with reduced paramedic time in the ED is based on the assumption that paramedics could be ‘mobile and active within the community’ if not waiting in the ED. That is, there is an opportunity cost associated with the paramedic waiting in the ED and, as such, a reduction in this waiting time is considered a cost offset.

Quantitative data were analysed using SPSS (IBM Corporation, Armonk, NY, USA) and Microsoft Excel (for the CEA). Statistical significance was set at *P* < 0.05.

### *Ethics*

The study received human research ethics committee approval from the Hospital and Health Service and Griffith University (HREC/12/QGC/190; NRS13/13/HREC). Approval was also received from QAS.

## Results

A total of 6045 people made 7010 presentations to the ED by ambulance over the study period. Patient demographics and clinical characteristics (age, gender, triage categories, top five International Classification of Diseases [ICD‐10] codes, shift of arrival, weekday/weekend presentation) did not differ significantly across the three study periods (Table 1).

**Table 1:** ED demographic and clinical characteristics for patient presentations made to ED via ambulance during the study period

Characteristic	Pre (T1) *n* = 2463 (%)	During (T2) *n* = 2348 (%)	Post (T3) *n* = 2199 (%)	*P*‐value
Median age in years (IQR)	51 (28–73)	50 (27–73)	48 (25–72)	0.05†
Gender: female	1203 (48.8%)	1159 (49.4%)	1078 (49.0%)	0.94‡
Triage category				0.28‡
ATS 1	73 (3.0%)	76 (3.2%)	56 (2.5%)	
ATS 2	681 (27.6%)	573 (24.4%)	572 (26.0%)	
ATS 3	1370 (55.6%)	1359 (57.9%)	1278 (58.1%)	
ATS 4	331 (13.4%)	332 (14.1%)	284 (12.9%)	
ATS 5	8 (0.3%)	8 (0.3%)	9 (0.4%)	
ED ICD‐10 MDC				0.20‡
Trauma (S00‐T88)	531 (21.6%)	544 (23.2%)	494 (22.5%)	
Diseases of the circulatory system (I00–I99)	368 (14.9%)	297 (12.6%)	295 (13.4%)	
Diseases of the respiratory system (J00–J99)	180 (7.3%)	188 (8.0%)	143 (6.5%)	
Diseases of the neurological system (G00–G99)	181 (7.3%)	156 (6.6%)	161 (7.3%)	
Diseases of the digestive system (K00–K93)	147 (6.0%)	140 (6.0%)	154 (7.0%)	
All other	1056 (42.9%)	1023 (43.6%)	952 (43.3%)	
Shift of presentation				0.46‡
Morning (07.00–14.59 hours)	856 (34.8%)	860 (36.6%)	820 (37.3%)	
Evening (15.00–22.59 hours)	1035 (42.0%)	950 (40.5%)	885 (40.2%)	
Night (23.00–06.59 hours)	572 (23.2%)	538 (22.9%)	494 (22.5%)	
Weekday/weekend				0.09‡
Weekday	1742 (70.7%)	1594 (67.9%)	1539 (70.0%)	
Weekend	721 (29.3%)	754 (32.1%)	660 (30.0%)	

† *P*‐value based on Kruskal–Wallis one‐way analysis of variance.

‡ *P*‐value based on χ^2^ test.

ATS, Australasian Triage Scale; ICD‐10, International Statistical Classification of Diseases and Related Health Problems; IQR, interquartile range; MDC, major diagnostic categories.

Several processes of care measures improved significantly between T1 and T2 for ambulance arriving patient presentations (Table 2). These included median ambulance offload time (T1: 26 min; T2: 24 min), the proportion offloaded within 30 min (i.e. offload compliant) (T1: 58%; T2: 63%), time at ED (T1: 43 min; T2: 39 min), median ED LoS for all presentations (T1: 335 min; T2: 306 min), and the proportion of arrivals with an ED LoS <4 h (i.e. NEAT compliant) (T1: 33%; T2: 36%). Some of these improvements, such as median ambulance time at ED and ED LoS carried over into T3, albeit to a lesser extent.

**Table 2:** Processes of care measures for patient presentations made to ED via ambulance during the study period

Processes of care measure	Pre (T1)	During (T2)	Post (T3)	*P*‐value¶
	*n* = 2463	*n* = 2348	*n* = 2199	T1 *vs* T2, T2 *vs* T3, T1 *vs* T3
Median ambulance offload time (IQR), min**†**	26 (15–46)	24 (14–41)	24 (15–45)	<0.001, 0.10, 0.08
Median ambulance time at ED (IQR), min**†**	43 (29–67)	39 (26–61)	40 (26–62)	<0.001, 0.24, <0.001
Time at ED if arrived morning shift	41 (27–64)	38 (24–59)	39.5 (26–66)	0.01, 0.08, 0.44
Time at ED if arrived evening shift	47 (32–70)	42 (29–67)	43 (29–67)	0.004, 0.80, 0.01
Time at ED if arrived night shift	38 (26–60)	34 (24–50)	34 (23–50)	0.003, 0.756, 0.002
Ambulance offload time <30 min, *n* (%)**†**	1411 (57.5%)	1465 (62.5%)	1319 (60.1%)	<0.001, 0.10, 0.001
Median time to be seen by doctor, min (IQR)**‡**	24 (7–81)	22 (7–70)	24 (7–83)	0.38
Seen within ATS, *n* (%)**‡**	1162 (51.8%)	1188 (54.6%)	1058 (52.1%)	0.12
ED LoS <4 h, *n* (%)§	746 (33.2%)	784 (36.0)	635 (31.2)	0.05, 0.001, 0.005
Median ED LoS, min (*all*) (IQR)§	335 (200–524)	306 (195–483)	330 (206–503)	0.003, <0.02, 0.49
Median ED LoS, min (*admitted)* (IQR)§	407 (262–646)	397 (257–596)	394 (264–583)	0.22
Median ED LoS, min (*not admitted*) (IQR)§	253 (164–415)	241 (161–383)	267 (167–392)	0.16
Admitted to hospital, *n* (%)	1189 (48.3%)	1057 (45.0%)	1073 (48.8%)	0.025, 0.012, 0.745

† Analysis based on 6991 ED presentations (*n* = 8, 5 and 6 missing for T1, T2 and T3, respectively).

‡ Analysis based on 6448 ED presentations (219, 173 and 170 are missing during T1, T2 and T3, due to patients DNW or missing ‘time to be seen’ data).

§ Analysis based on 6457 ED presentations (missing data due patients who did not wait to be seen for T1: *n* = 216; T2: *n* = 170; T3: *n* = 167).

¶ Analysis based on Kruskal–Wallis 1‐way anova (k samples) and, where significant, pairwise post‐hoc Mann–Whitney *U*‐tests for continuous data and χ^2^ for categorical variables.

ATS, Australasian Triage Scale; ED LoS, ED length of stay; IQR, interquartile range.

After controlling for covariates (sex, age, triage code, major diagnostic condition, day of the week and shift), the EDAOLN was associated with a predicted 11 min reduction in time to be seen by doctor per presentation (*P* < 0.001; 95% CI 10.27–11.16) and a 19 min reduction in ED LoS per presentation (*P* = 0.018; 95% CI 3.28–34.78). It was also associated with a 4 percentage point increase in the proportion of presentations that were ATS compliant (*P* = 0.001; 95% CI 1.6–6.8) and a non‐statistically significant 1.5 percentage point increase in the proportion of presentations that were NEAT compliant (*P* = 0.269; 95% CI −1.2–4.2) (Table 3, Appendices S5 and S6).

**Table 3:** Analysis of cost and effects: EDAOLN (T2) *versus* pre‐EDAOLN (T1)

	Mean
Costs	
Total cost of EDAOLN (i.e. during T2)	$28 816
Ambulance	
Change in time per attendance, min (95% CI)	−5.78 (−2.10, −9.46)
Total cost offset	$15 230 ($5539, $24 921)
Net cost of EDAOLN	$13 586 ($3895, $23 277)
Effects	
Reduction in TTBS per attendance, min (95% CI)	10.72 (10.27, 11.16)
Reduction in ED LoS per attendance, min (95% CI)	19.03 (3.28, 34.78)
Increase in ATS compliance, percentage points (95% CI)	4.2% (1.6%, 6.8%)
Increase in NEAT compliance, percentage points (95% CI)	1.5% (−1.2%, 4.2%)

ATS, Australasian Triage Scale; ATS compliance, % of patients seen within recommended ATS timeframe; CI, credible interval; ED LoS, ED length of stay; NEAT, National Emergency Access Target; NEAT compliance, % of patients seen, admitted, discharged within 4 h of arrival; TTBS, time to be seen by doctor.

When considering patient presentations in terms of their urgency (urgent: ATS 1 and 2, less urgent: ATS 3, 4 and 5) post‐hoc sub‐group analyses showed that several processes of care measures differed. ED LoS was shorter and admission rate was lower in the less urgent patient presentations pre‐, during and post‐EDAOLN timeframes (Table 4). Offload time, offload compliance, time to be seen by a doctor, the proportion seen within ATS, and ED LoS <4 h were improved during the EDAOLN trial period (T2 compared to T1) for the ATS 3, 4, 5 group (Table 4). For the ATS 1 and 2 group, other than offload time (2 min shorter), no other outcomes improved significantly during the EDAOLN trial period.

**Table 4:** Processes of care measures for patient presentations made to ED via ambulance by ATS; urgent (ATS 1, 2) and less urgent (ATS 3, 4, 5)

Process of care measure	Pre (T1) ATS 1, 2 ATS 3, 4, 5		During (T2) ATS 1, 2 ATS 3, 4, 5		Post (T3) ATS 1, 2 ATS 3, 4, 5		*P*‐value§
Median ambulance offload time (IQR), min**†**	19 (10–33)	29 (18–51)	17 (9–30)	27 (16–46)	19 (10–33)	27 (17–50)	U: **0.02** (0.007, 0.03, 0.71); LU: **0.002** (<0.001, 0.39, 0.02)
Ambulance offload <30 min, *n* (%)**†**	536 (71.2%)	875 (51.4%)	494 (76.1%)	971 (57.3%)	448 (71.6%)	871 (55.6%)	U: 0.08; LU: **0.002**
Median time to be seen by a doctor, (IQR), min**‡**	6 (2–10)	54 (21–127)	5 (2–9)	44 (18–100)	5 (2–9)	54 (20–206)	U: 0.43; LU: **<0.001** (<0.001, 0.001, 0.42)
Seen within ATS, *n* (%)	616/752 (81.9%)	546/1492 (36.5%)	523/647 (80.8%)	665/1528 (43.5%)	519/627 (82.8%)	539/1402 (38.4%)	U: 067; LU: **<0.001**
ED LoS <4 h, *n* (%)**‡**	239/752 (31.8%)	513/1495 (34.3%)	210/647 (32.5%)	577/1531 (37.7%)	169/626 (27.0%)	472/1405 (33.6%)	U: 0.13; LU: **0.002**
ED LoS, min Median (IQR)	351 (207–353)	323 (198–521)	328 (206–502)	294 (190–474)	340 (232–502)	325 (198–495)	U: 0.32; LU: **0.03 (0.008, 0.08, 0.36)**
ED LoS, min (*admitted*) Median (IQR)	397 (250–616)	412 (271–662)	386 (259–594)	400 (257–605)	387 (278–559)	400 (256–609)	U: 0.74; LU: 0.25
ED LoS, min**†** (*not admitted*) Median (IQR)	261 (169–421)	251 (161–408)	248 (168–400)	239 (161–378)	269 (184–419)	265 (163–387)	U: 0.59; LU: 0.27
Admitted, *n* (%)	458 (60.7%)	731 (42.8%)	391 (62.3%)	666 (42.4%)	409 (63.0%)	664 (39.1%)	U: 0.13; LU: 0.07

† Analyses based on 6991 ED presentations.

‡ Analyses based on 6448 ED presentations (due to patients who did not wait/left against medical advice).

§ Analysis based on Kruskal–Wallis 1‐way anova.

ATS, Australasian Triage Scale; EDIS, emergency department information system; ED LoS, ED length of stay; IQR, interquartile range; OST, off‐stretcher time; U, urgent (ATS 1, 2); LU, less urgent (ATS 3, 4, 5).

Regarding crude ED episode of care costs, no significant differences were identified in the ED episode of care cost per patient presentation made by ambulance during each time period (T1: $789; T2: $789; T3: $803; *P* = 0.9) or when considered by shift (morning, evening, night) (Appendix S7).

The total cost of the EDAOLN for 39 days of T2 was $28 816 with the cost per shift varying from $224 (any weekday morning shift) to $449 (any Sunday shift). This cost was offset by a $15 230 (95% CI $5539–$24 921) theoretical cost offset from reduced ambulance staff waiting time in ED (ranging from an additional $197 per shift (Wednesday morning shift) to a cost offset of $524 (Saturday evening shift)). The net cost (i.e. cost of EDAOLN minus the cost offset from reduced ambulance staff waiting time) of the EDAOLN for 39 days was $13 586 (Table 3, table in Appendix S4 and Appendix S8). Overall, if funded as an additional position, rather than from existing resources, the EDAOLN would be associated with an additional net annualised cost of $130 721 ($13 586/39 days × 365.25) and an annualised reduction in waiting time to be seen by a doctor of 234 828 (10.72 min × 2339 presentations/39 days × 365.25) min or approximately 3912 h or 163 days. Where the EDAOLN position can be implemented within existing resources, and with no impact on other ED care processes, the annualised cost offset would be $142 635.

From the analysis at a day/shift level, the most cost‐effective shifts to implement the EDAOLN (in order of cost‐effectiveness) were Thursday morning, Saturday night, Friday night, Wednesday evening, Monday evening and Tuesday morning (Appendix S8). These shifts were all associated with cost offsets greater than the cost of the EDAOLN (although not statistically significant) and statistically significant reductions in time to be seen.

## Discussion

During the time the EDAOLN role was operational, modest improvements in some process measures (offload time, time to be seen, ED LoS) were noted. Access delays increase the risk of poor outcomes such as longer lengths of hospital stay.^8^ By reconfiguring existing staffing resources towards access into the ED, the temporary implementation of the EDAOLN role may enhance the ED journey for some patients arriving by ambulance by directly addressing transfer concerns.

Our results indicate that the reality of meeting policy mandates such as NEAT and POST are challenging in the clinical arena. Our study was conducted during the first year of the state‐wide implementation of NEAT where the target was set at 70%.^27^ During the study time period, 34% of patients arriving to the ED by ambulance met NEAT and 60% had a POST of <30 min. Difficulties reaching the NEAT are not unique to our study, with other studies (not limited to those arriving by ambulance) from 2012 reporting NEAT figures of 53%–54%.^28^, ^29^ Limited research exists regarding POST making comparison with our findings difficult. Earlier multi‐site research reported that for ambulance arriving patients (*n* = 40 783), 37% had an ED LoS of <4 h and 85% had a POST of <30 min.^8^ Although NEAT and POST outcomes improved during the time the EDAOLN was operational, further opportunities to improve access into and out of the ED warrant consideration. Broader organisational factors (such as limited inpatient beds to transfer people from the ED to) possibly explain why the magnitude of these process measures were not larger. At the time of the study, hospitals and ambulance had different governing bodies. Although this has since changed, with both now operating within the one state health department, issues around POST continue.^11^, ^12^

Our study found that the EDAOLN role was, as planned, most helpful for less urgent patients who, by virtue of their presenting condition and triage category, can experience longer waits in the ED than those deemed more urgent. The EDAOLN is not, however, the only potential solution to address ambulance access issues into the ED. In the United States, an evidence‐based toolkit was developed to assist hospitals with reducing ambulance offload delays.^30^ Other broader measures that may warrant consideration, implementation and evaluation (including economic analysis) include the allocation of other nurses with extended scope of practice or nurse practitioners to the triage area^20^, ^21^ and the development of a physician‐assisted triage process.^31^ ED avoidance models have also been developed where nurses accompany paramedics on low acuity ambulance calls to treat patients on site (i.e. at home).^32^, ^33^, ^34^

In addition, to minimise the extent of access block within the ED, opportunities outside the ED warrant consideration and implementation. Such strategies include: holding units, early discharge and patient flow, political action management, resource priority,^35^ increased inpatient bed capacity, improved coordination and capacity within the community to manage patients with complex medical conditions, and improvements with hospital processes.^36^ Essentially, a coordinated whole of health system coupled with increased and improved hospital and alternative care capacity and evidence‐informed strategies are required to address this most serious issue facing EDs.^37^

The economic evaluation of the EDAOLN trial indicates that the role was most beneficial at certain times and certain days, especially Monday evening, Friday night and Saturday night (well‐recognised times for ED busyness). This was also evident when ambulance data were analysed, which indicated reduced times (and therefore costs) for paramedics waiting in ED to offload patients. The EDAOLN role was also cost effective; producing clinically meaningful reductions in access parameters such as time to be seen for a modest cost. While other reports and studies describe similar roles,^22^, ^30^ accompanying comprehensive economic evaluations of their impact on ED process measures were not apparent.

There are several limitations of the present study. This was a single site study with data collected in 2012 (about 6 years prior this analysis) and findings may not be contemporary or generalisable to other EDs. The intervention was however, based on a clinical and service need during a time of unprecedented ED crowding, an issue that is still evident and not unique to the study site ED. The short duration of the trial may have limited the extent of outcomes seen. The timeframe was however pragmatic, stipulated by hospital managers, and based on clinical demand. Data collected for the present study was limited to the time periods before, during and after the implementation of the EDAOLN role. Without 12 months (or more) worth of data, we were unable to account for potential seasonal influences. The large sample size gives rise to very small findings (either differences or associations) identified as statistically significant, thus, when interpreting the results of the present study, clinical significance is also an important consideration. While we were able to account for some confounders, improvements in process measures seen during the trial period and following the trial period may reflect additional service improvement measures implemented of which we were unaware. The use of secondary data that is routinely entered in a prospective fashion by clinical and administrative staff may be subject to data entry errors. However the linkage between data sources (ED, ambulance and cost) strengthened our ability to understand aspects of patient flow for this group and provided more depth of data analysis than is possible from one source alone. Additional linkage with in‐patient data and care delivery documentation is recommended to enable analysis of other outcome measures (such as adverse events) not reported here. Data collection and linkage for the entire ED population is also suggested to capture potential unintended consequences on people who arrive to the ED by means other than ambulance. Finally, as this was a retrospective observational study we cannot infer a direct causal relationship between the EDAOLN role and patient/health service process measures; however, the trial was pragmatic, made the best use of real‐world data and the findings were promising suggesting it would be worth considering undertaking a rigorous trial of such a role.

## Conclusion

In the context of patient safety and patient flow, findings from the present study indicate that the EDAOLN role could be considered, alongside other hospital strategies, to support immediate care requirements for patients arriving to the ED by ambulance during noted times of ED busyness (i.e. Monday afternoon, Friday and Saturday nights). Findings from the present study can be used to inform further service development at a local and potentially state‐wide or national level, and lead to further work to evaluate this (or similar) roles at other sites. By incorporating further outcome metrics in future research (such as adverse events, hospital LoS, hospital mortality) there is opportunity to investigate certain sub‐groups of ED presenters who may benefit most from the EDAOLN role.

## Supporting information


**Appendix S1.** An overview of the EDAOLN role.

**Appendix S2.** Summary of data collection sources and description, data collected, purpose and data linkage strategy.

**Appendix S3.** Definitions of processes of care measures used in other studies or reports.

**Appendix S4.** Details informing cost effectiveness analysis.

**Appendix S5.** Results for regression analysis of ambulance service time.

**Appendix S6.** Results for regression analysis of time to be seen by ED doctor.

**Appendix S7.** Episode of care costs for patient presentations made to ED via ambulance, by shift and study period.

**Appendix S8.** Cost‐effectiveness analysis (time to be seen): EDAOLN (T2) vs pre‐EDAOLN (T1) by shift.

## References

[1] Australian Institute of Health and Welfare . Australian Hospital Statistics 2016–17: Emergency Department Care. 2017. Contract No.: Health services series no. 80. Cat. no. HSE 194.

[2] Australian Institute of Health and Welfare . Australian Hospital Statistics 2010–11. 2012. Contract No.: Health Services Series no.43. Cat. no. HSE 117.

[3] Lecky F , Benger J , Mason S *et al* The International Federation for Emergency Medicine framework for quality and safety in the emergency department. Emerg. Med. J. 2014; 31: 926–9. 23907367 10.1136/emermed-2013-203000

[4] Hitchcock M , Crilly J , Gillespie B , Chaboyer W , Tippett V . The effect of ambulance ramping on emergency department length of stay and in‐patient mortality. Aust. Emerg. Nurs. J. 2010; 13: 17–24.

[5] Burke LG , Joyce N , Baker WE *et al* The effect of an ambulance diversion ban on emergency department length of stay and ambulance turnaround time. Ann. Emerg. Med. 2013; 61: 303–11 e1. 23352752 10.1016/j.annemergmed.2012.09.009

[6] Kingswell C , Shaban R , Crilly J . Concepts, antecedents and consequences of ambulance ramping in the emergency department: a scoping review. Australas. Emerg. Nurs. J. 2017; 20: 153–60. 29054574 10.1016/j.aenj.2017.07.002

[7] Cooney DR , Wojcik S , Seth N , Vasisko C , Stimson K . Evaluation of ambulance offload delay at a university hospital emergency department. Int. J. Emerg. Med. 2013; 6: 15. 23663387 10.1186/1865-1380-6-15 PMC3663714

[8] Crilly J , Keijzers G , Tippett V *et al* Improved outcomes for emergency department patients whose ambulance off‐stretcher time is not delayed. Emerg. Med. Australas. 2015; 27: 216–24. 25940975 10.1111/1742-6723.12399 PMC4676924

[9] Cooney DR , Millin MG , Carter A , Lawner BJ , Nable JV , Wallus HJ . Ambulance diversion and emergency department offload delay: resource document for the National Association of EMS physicians position statement. Prehosp. Emerg. Care 2011; 15: 555–61. 21870947 10.3109/10903127.2011.608871

[10] Carter AJ , Gould JB , Vanberkel P *et al* Offload zones to mitigate emergency medical services (EMS) offload delay in the emergency department: a process map and hazard analysis. Canadian J. Emerg. Med. 2015; 17: 670–8. 10.1017/cem.2015.15 25994045

[11] Carter AJ , Overton J , Terashima M , Cone DC . Can emergency medical services use turnaround time as a proxy for measuring ambulance offload time? J. Emerg. Med. 2014; 47: 30–5. 24373216 10.1016/j.jemermed.2013.08.109

[12] Kingswell C , Shaban R , Crilly J . The lived experiences of patients and ambulance ramping in a regional Australian emergency department: an interpretive phenomenology study. Australas. Emerg. Nurs. J. 2015; 18: 182–9. 26603895 10.1016/j.aenj.2015.08.003

[13] Greaves T , Mitchell M , Crilly J . The impact of an emergency department ambulance offload nurse role: a retrospective study. Int. Emerg. Nurs. 2017; 32: 39–44. 28285102 10.1016/j.ienj.2016.12.005

[14] Australasian College for Emergency Medicine . *Position Statement on Ambulance Ramping (Also Known as Off‐Stretcher or Ambulance Turnaround Delays)* 2018. [Cited March 2019.] Available from URL: https://acem.org.au/getmedia/9e6c3e78-8cbc-473c-83df-474f6c1eecde/S347-Statement-on-Ambulance-Ramping-Nov-13.aspx

[15] Department of Health . *A&E Clinical Quality Indicators Implementation Guidance* [Cited March 2019.] Available from URL: http://webarchive.nationalarchives.gov.uk/20130107105354/ http://http:/www.dh.gov.uk/prod_consum_dh/groups/dh_digitalassets/@dh/@en/@ps/documents/digitalasset/dh_123055.pdf

[16] Millin MG , Brown LH , Schwartz B . EMS provider determinations of necessity for transport and reimbursement for EMS response, medical care, and transport: combined resource document for the National Association of EMS physicians position statements. Prehosp. Emerg. Care 2011; 15: 562–9. 21797787 10.3109/10903127.2011.598625

[17] Bost N , Crilly J , Wallis M , Patterson E , Chaboyer W . Clinical handover of patients arriving by ambulance to the emergency department ‐ a literature review. Int. Emerg. Nurs. 2010; 18: 210–20. 20869662 10.1016/j.ienj.2009.11.006

[18] Bost N , Crilly J , Patterson E , Chaboyer W . Clinical handover of patients arriving by ambulance to a hospital emergency department: a qualitative study. Int. Emerg. Nurs. 2012; 20: 133–41. 22726945 10.1016/j.ienj.2011.10.002

[19] Australian Commission on Safety and Quality in Health Care . *Australian Safety and Quality Framework for Health Care* 2010. [Cited March 2019.] Available from URL: https://www.safetyandquality.gov.au/wp-content/uploads/2012/04/Australian-SandQ-Framework1.pdf

[20] Crawford K , Morphet J , Jones T , Innes K , Griffiths D , Williams A . Initiatives to reduce overcrowding and access block in Australian emergency departments: a literature review. Collegian 2014; 21: 359–66. 25632734 10.1016/j.colegn.2013.09.005

[21] Love RA , Murphy JA , Lietz TE , Jordan KS . The effectiveness of a provider in triage in the emergency department: a quality improvement initiative to improve patient flow. Adv. Emerg. Nurs. J. 2012; 34: 65–74. 22313903 10.1097/TME.0b013e3182435543

[22] Considine J , Lucas E , Payne R , Kropman M , Stergiou HE , Chiu H . Analysis of three advanced practice roles in emergency nursing. Aust. Emerg. Nurs. J. 2012; 15: 219–28. 10.1016/j.aenj.2012.10.001 23217655

[23] Donabedian A , Wheeler JR , Wyszewianski L . Quality, cost, and health: an integrative model. Med. Care 1982; 20: 975–92. 6813605 10.1097/00005650-198210000-00001

[24] Irvine D , Sidani S , Hall M . Linking outcomes to nurses' role in health care. Nurs. Econ. 1998; 16: 58–64. 9592519

[25] Australian Bureau of Statistics . *3218.0 Regional Population Growth, Australia, 2012–2013* 2018 [Cited April 2019.] Available from URL: http://www.abs.gov.au/AUSSTATS/abs@.nsf/Previousproducts/3218.0Media%20Release12012-13

[26] Australasian College for Emergency Medicine . Guidelines on the implementation of the Australasian Triage Scale in emergency departments. [Cited April 2019.] Available from URL: https://acem.org.au/getmedia/51dc74f7-9ff0-42ce-872a-0437f3db640a/G24_04_Guidelines_on_Implementation_of_ATS_Jul-16.aspx

[27] Council of Australian Governments . *The National Health Reform Agreement ‐ National Partnership Agreement on Improving Public Hospital Services* 2011. [Cited 28 Mar 2019.] Available from URL: http://www.federalfinancialrelations.gov.au/content/npa/health/_archive/national-workforce-reform/national_partnership.pdf

[28] Bost N , Crilly J , Wallen K . Characteristics and process outcomes of patients presenting to an Australian emergency department for mental health and non‐mental health diagnoses. Int. Emerg. Nurs. 2014; 22: 146–52. 24439293 10.1016/j.ienj.2013.12.002

[29] Perera ML , Davies AW , Gnaneswaran N *et al* Clearing emergency departments and clogging wards: National Emergency Access Target and the law of unintended consequences. Emerg. Med. Australas. 2014; 26: 549–55. 25332129 10.1111/1742-6723.12300

[30] Bartleson BJ , Backer H . Toolkit to reduce ambulance patient offload delays in the emergency department In: Building Strategies for California Hospitals and Local Emergency Services Agencies. Sacramento, CA: Californian Hospitals Association, 2014.

[31] Elder E , Johnston ANB , Crilly J . Systematic review of three key strategies designed to improve patient flow through the emergency department. Emerg. Med. Australas. 2015; 27: 394–404. 26206428 10.1111/1742-6723.12446

[32] Sjolin H , Lindstrom V , Hult H , Ringsted C , Kurland L . What an ambulance nurse needs to know: a content analysis of curricula in the specialist nursing programme in prehospital emergency care. Int. Emerg. Nurs. 2015; 23: 127–32. 25304861 10.1016/j.ienj.2014.09.002

[33] Tohira H , Williams TA , Jacobs I , Bremner A , Finn J . The impact of new prehospital practitioners on ambulance transportation to the emergency department: a systematic review and meta‐analysis. Emerg. Med. J. 2014; 31: e88–94. 24243486 10.1136/emermed-2013-202976

[34] Machen I , Dickinson A , Williams J , Widiatmoko D , Kendall S . Nurses and paramedics in partnership: perceptions of a new response to low‐priority ambulance calls. Accid. Emerg. Nurs. 2007; 15: 185–92. 17993276 10.1016/j.aaen.2007.09.001

[35] Chan SS , Cheung NK , Graham CA , Rainer TH . Strategies and solutions to alleviate access block and overcrowding in emergency departments. Hong Kong Med. J. 2015; 21: 343–52. 10.12809/hkmj144399 26087756

[36] Cameron PA , Joseph AP , McCarthy SM . Access block can be managed. Med. J. Aust. 2009; 190: 364–8. 19351310 10.5694/j.1326-5377.2009.tb02449.x

[37] Australasian College for Emergency Medicine . *Access Block Position Statement S127* 2019 [Cited 15 Oct 2019.] Available from URL: https://acem.org.au/

