# Supplementary material for: Improving emergency department transfer for patients arriving by ambulance: A retrospective observational study
Source: Emerg Med Australas. 2019 Dec 23;32(2):271–80. doi: 10.1111/1742-6723.13407 (PMC7155107; doi:10.1111/1742-6723.13407)
Supplement: Supplementary file 1 — Appendix S1. An overview of the EDAOLN role. [file EMM-32-271-s001.doc]

**Appendix S1. An overview of the EDAOLN role**

Interviews with key stakeholders from the ED and ambulance service were undertaken to inform the description of the EDAOLN role. The role was operationalised by a temporary reconfiguration of existing nursing staffing. The aim of the EDAOLN role was to expedite ambulance-ED transfer, particularly for patients triaged with an Australasian Triage Scale (ATS) of 3, 4 or 5 and waiting for an ED bed. This scope was relevant as ATS 1 and 2 patients were likely to be offloaded onto an ED bed soon after arrival. The role of the EDAOLN included rapid triage and assessment (including recording of patients’ observations such as heart, rate, blood pressure, blood sugar level), and commencement of initial meaningful treatment (such as initiating or arranging X-rays, pathology, analgesia) as required. Nurses working in the EDAOLN role were required to be triage competent, with quality assessment skills, able to recognise patient deterioration, problem-solve, and communicate well. Three chairs were specifically allocated for EDAOLN use, located in view of ED staff and near the ambulance entrance. This physical space was required to facilitate expedited access to initial assessment and treatment by the EDAOLN and enabled the release of paramedics to respond back into the community.
