# Supplementary material for: Improving emergency department transfer for patients arriving by ambulance: A retrospective observational study
Source: Emerg Med Australas. 2019 Dec 23;32(2):271–80. doi: 10.1111/1742-6723.13407 (PMC7155107; doi:10.1111/1742-6723.13407)
Supplement: Supplementary file 2 — Appendix S2. Summary of data collection sources and description, data collected, purpose and data linkage strategy. [file EMM-32-271-s002.doc]

**Appendix S2. Summary of data collection sources and description, data collected, purpose and data linkage strategy**

| **Data source and description** | **Data collected** | **Purpose of data collection** |
| --- | --- | --- |
| QAS database: routinely and prospectively collected data by paramedics regarding the patients episode of care involving ambulance dispatch and response | Name, age, sex, postcode pick up, suburb pick up, Communication Centre triage code, suburb location of base station, date, and times of dispatch, site arrival, site departure, arrival at ED, triage by ED, stretcher off-load, departure from ED, off-load destination | To describe patient and health service outcomes of the EDAOLN role |
| ED database: routinely and prospectively collected data by ED staff (administrative and clinical) regarding the patients ED episode of care | Medical unit record (URN) number, name, date of birth, sex, post code, reason for ED presentation, ED diagnosis (ICD code), mode of arrival, ATS category, date & time of presentation, date & time of ED triage, date & time seen by doctor, date & time of departure from ED, discharge destination from ED | To describe patient and health service outcomes of the EDAOLN role |
| Transitions II database: patient level cost data attributed to care and treatments provided during the ED episode of care, assigned by trained health service cost/ finance staff | Medical unit record (URN) number, name, date of birth, sex, cost of ED episode of care | To describe health economic impact of the EDAOLN role |

Abbreviations: ATS: Australasian Triage Scale; ED: Emergency Department; EDAOLN: ED ambulance offload nurse; ICD:

International Classification of Diseases and Related Health Problems; QAS: Queensland Ambulance Service; URN: Unit Record Number

Data from each of database were deterministically linked using Commonwealth Scientific and Industrial Research Organization’s Health

Data Integration (HDI) software^1^ which was refined with input from clinicians and researchers. Once linkage was complete, 100 linked

EDIS and TII data records and 100 eARF records were randomly selected. These records were manually linked by one of the

researchers (AJ) to confirm the relative accuracy of the HDI linkage. An initial 15% error rate (EDIS to eARF) revealed an early linkage

error (around the fuzzy logic used to support name field linking) which, once resolved, dropped the error rate to 1%.

**Reference**

1. Hansen D, Pang C, Maeder A. HDI: Integrating Health Data and Tools. *Soft Computing*. 2007;**11**:361-7.
