# Supplementary material for: Improving emergency department transfer for patients arriving by ambulance: A retrospective observational study
Source: Emerg Med Australas. 2019 Dec 23;32(2):271–80. doi: 10.1111/1742-6723.13407 (PMC7155107; doi:10.1111/1742-6723.13407)
Supplement: Supplementary file 3 — Appendix S3. Definitions of processes of care measures used in other studies or reports. [file EMM-32-271-s003.doc]

**Appendix S3. Definitions of processes of care measures used in other studies or reports**

| **Process of care measure** | **Definition** |
| --- | --- |
| Ambulance patient off stretcher time (POST) | Time between arrival at the ED in an ambulance and logged transfer into an ED bed ^1,2^ |
| Ambulance offload compliance | Compliance (percentage) with the time target (30mins) from arrival in ED by ambulance to logged transfer into an ED bed ^1,3,4,5^ |
| Ambulance at ED turnaround time | Time elapsed between logged ambulance arrival at ED and logged ambulance departure from ED ^1,4,6^ |
| Time to be seen/treated in ED | Time elapsed between arrival/triage time and time seen by a treating doctor ^1,4^ |
| Seen within recommended ATS timeframe | Compliance (percentage) with the ATS time to be seen target ^1^ |
| ED length of stay | Duration of stay in the ED from arrival to departure ^1,4,5,7^ |
| NEAT compliance | Compliance (percentage) with the NEAT timeframe; i.e. patient transferred, admitted or discharged from the ED within 4 hours ^1,5,8^ |
| Admission rate | Proportion of ED patients admitted to hospital ^1,5^ |

ATS, Australasian Triage Scale; ED, emergency department; NEAT, national emergency access target;

POST, patient off stretcher time.

**References**

1. Crilly J, Keijzers G, Tippett V, O'dwyer J, Lind J, Bost N, et al. Improved outcomes for emergency

department patients whose ambulance off‐stretcher time is not delayed. *Emerg Med Australas*. 2015;**27**:216

24.

2. Cooney DR, Millin MG, Carter A, Lawner BJ, Nable JV, Wallus HJ. Ambulance diversion and

emergency department offload delay: resource document for the National Association of EMS Physicians

position statement. *Prehosp Emerg Care*. 2011;**15**:555-61.

3. Cooney DR, Wojcik S, Seth N, Vasisko C, Stimson K. Evaluation of ambulance offload delay at a university hospital emergency department. *Int J Emerg Med*. 2013;**6**:15.

4. Kingswell C, Shaban R, Crilly J. The lived experiences of patients and ambulance ramping in a regional Australian emergency department: an interpretive phenomenology study. *Australasian Emergency Nursing Journal*. 2015,18(4):182-189.

5. Greaves T, Mitchell M, Crilly J. The impact of an Emergency Department Ambulance offload nurse role: a retrospective study. *International Emergency Nursing* 2017, 32: 39-44.

6. Carter AJ, Overton J, Terashima M, Cone DC. Can emergency medical services use turnaround time as a proxy for measuring ambulance offload time? *J Emerg Med*. 2014;**47**:30-5.

7. Hitchcock M, Crilly J, Gillespie B, Chaboyer W, Tippett V, J. L. The effect of ambulance ramping on emergency department length of stay and in-patient mortality. *Aust Emerg Nurs J* 2010;**13**:17-24.

8.Council of Australian Governments. The National Health Reform Agreement - National Partnership Agreement on Improving Public Hospital Services. 2011. [Cited 28 March 2019] Available from URL:

http://www.federalfinancialrelations.gov.au/content/npa/health/_archive/national-workforce-reform/national_partnership.pdf (Accessed March 2019)
