# Supplementary material for: Improving emergency department transfer for patients arriving by ambulance: A retrospective observational study
Source: Emerg Med Australas. 2019 Dec 23;32(2):271–80. doi: 10.1111/1742-6723.13407 (PMC7155107; doi:10.1111/1742-6723.13407)
Supplement: Supplementary file 4 — Appendix S4. Details informing cost effectiveness analysis. [file EMM-32-271-s004.doc]

**Appendix S4. Details informing cost effectiveness analysis**

Costs to the hospital ED and ambulance service compared during-EDAOLN with pre-EDAOLN data. The primary effectiveness variable for the Cost Effectiveness Analysis (CEA) was time to be seen (a key patient-related process measure) with additional analyses based on ED LoS, percentage increase in both ATS and NEAT compliance. Results from the CEA are presented as incremental cost-effectiveness ratios (ICER). The ICER is the average additional cost associated with one additional unit of the measure of effect, and calculated as the cost for the intervention group minus the costs for the control group divided by the effects for the intervention group minus the effect for the control group.^1^ Uncertainty in the ICERs was estimated using Monte Carlo simulation of 1,000 draws to provide a 95% credible interval. To estimate the incremental change in continuous effectiveness outcomes, a generalised linear model (model specification assessed based on modified Parks test and Pregibon link test) was developed with the categorical variable of whether the presentation was during-EDAOLN or pre-EDAOLN. A logistic regression model was used for categorical outcome variables.

Economic analyses controlled for sex, age, triage code, major diagnostic condition, day of the week and shift. Interactions between the EDAOLN or pre-EDAOLN and the day of the week and shift were also included to identify any potential differential effect of the EDAOLN. Net costs of the intervention were based on the cost of a triage nurse per shift (including shift allowances) and allowed for cost offsets with respect to reduced paramedic time based on the salary of a paramedic (including shift allowances). Nursing cost was estimated as the cost per shift multiplied by total number of shifts during the EDAOLN period. Paramedic time reduced was estimated as the total ambulance staff time reduced (attendances during evaluation period x mean number of ambulance staff per attendance x estimated reduction in ambulance staff time per attendance during the EDAOLN compared to the pre-EDAOLN period x shift specific ambulance staff cost per minute x proportion of total attendances per shift).

Assumptions in the economic analysis included that the QAS and nurse shift times are similar, that the average cost for a triage nurse (Nurse grade 5 / level 3) was approximately $56,817 per annum^2^ and the base wage for ‘paramedic’ was $52,827 per annum^3^ and that “full-time” constituted 76 hours per fortnight. Annual wage rates reflect salaries close to study time frame. They excluded overtime, broken meals and other allowances that are highly variable. Shift allowances were included as base wage plus: 12.5% for afternoon shift; 15% for night shift: 50% for a Saturday shift, and 100% (double time) for a Sunday shift.^4^

**Appendix S4 Table. Results of Cost-Effectiveness Analysis: EDAOLN (T2) vs pre-EDAOLN (T1)**

|  | Mean | ICER (95%CI) |
| --- | --- | --- |
| Total Cost of EDAOLN (i.e. during T2) | $28,816 |  |
| Ambulance |  |  |
| Change in time per attendance, min (95% CI) | -5.78  (-2.10, -9.46) |  |
| Total Cost offset | $15,230  ($5,539, $24,921) |  |
| **Net cost of EDAOLN** | **$13,586**  ($3,895, $23,277) |  |
| Reduction in TTBS per attendance, mins (95% CI) | 10.72  (10.27, 11.16) | $0.54  ($0.22, $0.81) |
| Reduction in ED LoS per attendance, mins (95% CI) | 19.03  (3.28, 34.78) | $0.31  ($0.07, $1.24) |
| Increase in ATS compliance, percentage points (95% CI) | 4.2%  (1.6%, 6.8%) | $138.29  ($37.56, $437.03) |
| Increase in NEAT compliance, percentage points (95% CI) | 1.5%  (-1.2%, 4.2%) | $384.53  ($61.86, $1,784.24) |

CI: credible interval; ED: Emergency Department; ED LoS: ED Length of Stay; ICER: incremental cost effectiveness ratio; TTBS: time to be seen by doctor; ATS: Australasian Triage Scale; NEAT: National ED access time; ATS compliance: % of patients seen within recommended ATS timeframe; NEAT compliance: % of patients seen, admitted, discharged within 4hrs of arrival.

**References**

# **1. Drummond MF, Sculpher MJ, Torrance GW, O'Brien BJ, Stoddart GL. Methods for the Economic Evaluation of Health Care Programmes. Oxford University Press, 2005.**

2. QIRC. Queensland Health Nurses and Midwives award - State 2011. Industrial Relations Act 1999 - s.

130 - award review. Queensland Industrial Relations Commission 2012.

3. QIRC. Ambulance service employees' award State 2003 2012 Industrial Relations Act 1999 - s. 130 –

award review. Queensland Industrial Relations Commission 2012.

4. QIRC. Queensland ambulance service determination 2013. Queensland Industrial Relations Commission

2013.
