# Supplementary material for: Improving emergency department transfer for patients arriving by ambulance: A retrospective observational study
Source: Emerg Med Australas. 2019 Dec 23;32(2):271–80. doi: 10.1111/1742-6723.13407 (PMC7155107; doi:10.1111/1742-6723.13407)
Supplement: Supplementary file 5 — Appendix S5. Results for regression analysis of ambulance service time. [file EMM-32-271-s005.doc]

| **Appendix S5. Results for regression analysis of ambulance service time** | | | | | |
| --- | --- | --- | --- | --- | --- |
| **Ambulance Service time** | **Coef.** | **Std. Err.** | **P-value** | **[95% Conf.** | **Interval]** |
| Pre During Post |  |  |  |  |  |
| Pre | (Ref.) |  |  |  |  |
| During | -0.429 | 0.150 | 0.004 | -0.722 | -0.136 |
| Post | -0.194 | 0.143 | 0.175 | -0.475 | 0.086 |
|  |  |  |  |  |  |
| Sex |  |  |  |  |  |
| Male | (Ref.) |  |  |  |  |
| Female | 0.005 | 0.026 | 0.858 | -0.047 | 0.057 |
|  |  |  |  |  |  |
| Age (years) | 0.003 | 0.001 | 0.000 | 0.002 | 0.004 |
|  |  |  |  |  |  |
| Triage code |  |  |  |  |  |
| Immediate | (Ref.) |  |  |  |  |
| Urgent<10min | -0.277 | 0.081 | 0.001 | -0.435 | -0.119 |
| Semi-urgent<30min | -0.299 | 0.079 | 0.000 | -0.454 | -0.143 |
| Acute<60min | -0.509 | 0.086 | 0.000 | -0.677 | -0.341 |
| Non-urgent<120min | -0.993 | 0.236 | 0.000 | -1.456 | -0.530 |
|  |  |  |  |  |  |
| MDCTOP5 |  |  |  |  |  |
| Trauma | (Ref.) |  |  |  |  |
| Cardiovascular | -0.101 | 0.049 | 0.042 | -0.197 | -0.004 |
| Respiratory | -0.061 | 0.057 | 0.287 | -0.173 | 0.051 |
| Neurological | -0.001 | 0.057 | 0.985 | -0.113 | 0.110 |
| Gastro-Intestinal | -0.087 | 0.060 | 0.144 | -0.204 | 0.030 |
| Other | -0.060 | 0.034 | 0.081 | -0.127 | 0.007 |
|  |  |  |  |  |  |
| Shift |  |  |  |  |  |
| Morning | (Ref.) |  |  |  |  |
| Evening | 0.054 | 0.145 | 0.710 | -0.230 | 0.338 |
| Night | 0.088 | 0.175 | 0.614 | -0.255 | 0.432 |
|  |  |  |  |  |  |
| Day of week |  |  |  |  |  |
| Monday | -0.065 | 0.142 | 0.648 | -0.343 | 0.213 |
| Saturday | -0.101 | 0.146 | 0.492 | -0.387 | 0.186 |
| Sunday | -0.327 | 0.149 | 0.028 | -0.620 | -0.035 |
| Thursday | -0.157 | 0.143 | 0.273 | -0.438 | 0.124 |
| Tuesday | -0.034 | 0.140 | 0.806 | -0.308 | 0.239 |
| Wednesday | -0.120 | 0.147 | 0.415 | -0.409 | 0.168 |
|  |  |  |  |  |  |
| Constant | 4.440 | 0.132 | 0.000 | 4.181 | 4.699 |

Generalized linear model (Gamma family, log link); MDC: Major diagnostic Category
