# Supplementary material for: Improving emergency department transfer for patients arriving by ambulance: A retrospective observational study
Source: Emerg Med Australas. 2019 Dec 23;32(2):271–80. doi: 10.1111/1742-6723.13407 (PMC7155107; doi:10.1111/1742-6723.13407)
Supplement: Supplementary file 6 — Appendix S6. Results for regression analysis of time to be seen by ED doctor. [file EMM-32-271-s006.doc]

| **Appendix S6. Results for regression analysis of time to be seen by ED doctor** | | | | | |
| --- | --- | --- | --- | --- | --- |
| **Time to be seen** | **Coef.** | **Std. Err.** | **P-value** | **[95% Conf.** | **Interval]** |
| Pre During Post | |  |  |  |  |
| Pre | (Ref.) |  |  |  |  |
| During | 0.194 | 0.024 | 0.000 | 0.147 | 0.241 |
| Post | 0.170 | 0.024 | 0.000 | 0.124 | 0.216 |
|  |  |  |  |  |  |
| Sex |  |  |  |  |  |
| Male | (Ref.) |  |  |  |  |
| Female | 0.062 | 0.003 | 0.000 | 0.055 | 0.068 |
|  |  |  |  |  |  |
| Age (Years) | 0.003 | 0.000 | 0.000 | 0.003 | 0.004 |
|  |  |  |  |  |  |
| Triage code |  |  |  |  |  |
| Immediate | (Ref.) |  |  |  |  |
| Urgent<10min | 7.420 | 1.000 | 0.000 | 5.460 | 9.380 |
| Semi-urgent<30min | 9.624 | 1.000 | 0.000 | 7.664 | 11.584 |
| Acute<60min | 9.931 | 1.000 | 0.000 | 7.971 | 11.890 |
| Non-urgent<120min | 9.533 | 1.001 | 0.000 | 7.572 | 11.495 |
|  |  |  |  |  |  |
| MDC TOP5 |  |  |  |  |  |
| Trauma | (Ref.) |  |  |  |  |
| Cardiovascular | -0.006 | 0.008 | 0.438 | -0.021 | 0.009 |
| Respiratory | -0.008 | 0.007 | 0.285 | -0.022 | 0.007 |
| Neurological | 0.099 | 0.007 | 0.000 | 0.084 | 0.113 |
| Gastro-Intestinal | 0.185 | 0.007 | 0.000 | 0.172 | 0.198 |
| Other | 0.152 | 0.004 | 0.000 | 0.144 | 0.160 |
|  |  |  |  |  |  |
| Shift |  |  |  |  |  |
| Morning | (Ref.) |  |  |  |  |
| Evening | 0.491 | 0.023 | 0.000 | 0.447 | 0.536 |
| Night | 0.845 | 0.024 | 0.000 | 0.798 | 0.891 |
|  |  |  |  |  |  |
| Day of week |  |  |  |  |  |
| Monday | 0.529 | 0.022 | 0.000 | 0.485 | 0.573 |
| Saturday | 0.146 | 0.024 | 0.000 | 0.100 | 0.193 |
| Sunday | 0.168 | 0.024 | 0.000 | 0.120 | 0.216 |
| Thursday | 0.290 | 0.023 | 0.000 | 0.244 | 0.335 |
| Tuesday | 0.457 | 0.022 | 0.000 | 0.415 | 0.500 |
| Wednesday | 0.129 | 0.024 | 0.000 | 0.082 | 0.176 |
|  |  |  |  |  |  |
| Constant | -6.196 | 1.000 | 0.000 | -8.156 | -4.235 |

Generalized linear model (Poisson family, log link); ED: Emergency Department; MDC: Major diagnostic Category
