# Supplementary material for: Improving emergency department transfer for patients arriving by ambulance: A retrospective observational study
Source: Emerg Med Australas. 2019 Dec 23;32(2):271–80. doi: 10.1111/1742-6723.13407 (PMC7155107; doi:10.1111/1742-6723.13407)
Supplement: Supplementary file 7 — Appendix S7. Episode of care costs for patient presentations made to ED via ambulance, by shift and study period. [file EMM-32-271-s007.doc]

Appendix S7. Episode of care costs for patient presentations made to ED via ambulance, by shift and study period

| **ED costs** | **Pre (T1)** | **During (T2)** | **Post (T3)** |  |
| --- | --- | --- | --- | --- |
|  | **n= 2,453** | **n= 2,339** | **n= 2,192** | **†P value** |
| Total ED episode of care cost  Mean +/- SD | 789±442 | 789±449 | 803±437 | 0.900 |
| ED episode of care cost,  arrived morning shift  Mean +/- SD | 841±454 | 864±499 | 844±424 | 0.549 |
| ED episode of care cost,  arrived evening shift  Mean +/- SD | 745±424 | 788±426 | 801±454 | 0.403 |
| ED episode of care cost,  arrived night shift  Mean +/- SD | 790±446 | 749±411 | 741±420 | 0.096 |

ED: Emergency Department; ED LoS: ED Length of Stay; SD: standard deviation

† Analysis based on Generalised Linear Model Gamma-family, Log-link
