# Supplementary material for: Improving emergency department transfer for patients arriving by ambulance: A retrospective observational study
Source: Emerg Med Australas. 2019 Dec 23;32(2):271–80. doi: 10.1111/1742-6723.13407 (PMC7155107; doi:10.1111/1742-6723.13407)
Supplement: Supplementary file 8 — Appendix S8. Cost‐effectiveness analysis (time to be seen): EDAOLN (T2) vs pre‐EDAOLN (T1) by shift. [file EMM-32-271-s008.doc]

**Appendix S8. Cost-effectiveness analysis (time to be seen): EDAOLN (T2) vs pre-EDAOLN (T1) by shift**

|  |  | **MONDAY** | **TUESDAY** | **WEDNESDAY** | **THURSDAY** | **FRIDAY** | **SATURDAY** | **SUNDAY** |
| --- | --- | --- | --- | --- | --- | --- | --- | --- |
| **Morning** | Cost per shift ($) | 224 | 224 | 224 | 224 | 224 | 337 | 449 |
|  | Annual cost of shift | 11672 | 11672 | 11672 | 11672 | 11672 | 17508 | 23344 |
|  | Cost offset per shift ($) | -183 | -225 | 197 | -243 | -459 | -245 | -96 |
|  | Annual cost offset of shift | -9541 | -11724 | 10250 | -12658 | -23862 | -12748 | -4996 |
|  | Attendances per shift | 24 | 26 | 23 | 26 | 23 | 23 | 30 |
|  | Incremental cost ($) | 410 | -1 | 422 | -19 | -234 | 92 | 353 |
|  | Incremental reduction (minutes) | 446 | 590 | -73 | 39 | -153 | -342 | 171 |
|  | ICER (95% CI) | **0.09** | **Dominant** | **Dominated** | **Dominant** | **nc** | **Dominated** | **2.06** |
|  |  | **(-0.60, 0.84)** | **(-0.71, 0.69)** |  | **(-25.23, 23.00)** |  |  | **(-1.65, 6.63)** |
| **Evening** | Cost per shift ($) | 253 | 253 | 253 | 253 | 253 | 337 | 449 |
|  | Annual cost of shift | 13131 | 13131 | 13131 | 13131 | 13131 | 17508 | 23344 |
|  | Cost offset per shift ($) | -259 | -2 | -326 | -211 | -226 | -524 | -46 |
|  | Annual cost offset of shift | -13446 | -106 | -16966 | -10983 | -11776 | -27251 | -2387 |
|  | Attendances per shift | 27 | 26 | 28 | 26 | 28 | 27 | 36 |
|  | Incremental cost ($) | -6 | 250 | -74 | 41 | 26 | -187 | 403 |
|  | Incremental reduction (minutes) | 1,374 | 562 | 437 | -686 | -90 | -43 | 403 |
|  | ICER (95% CI) | **Dominant** | **0.45** | **Dominant** | **Dominated** | **Dominated** | **nc** | **1** |
|  |  | **(-0.26, 0.24)** | **(-0.26, 1.14)** | **(-1.25, 0.74)** |  |  |  | **(-0.87, 2.84)** |
| **Night** | Cost per shift ($) | 258 | 258 | 258 | 258 | 258 | 337 | 449 |
|  | Annual cost of shift | 13423 | 13423 | 13423 | 13423 | 13423 | 17508 | 23344 |
|  | Cost offset per shift ($) | 73 | -209 | -28 | -39 | -283 | -386 | -292 |
|  | Annual cost offset of shift | 3773 | -10870 | -1470 | -2014 | -14722 | -20056 | -15173 |
|  | Attendances per shift | 16 | 12 | 13 | 12 | 14 | 18 | 28 |
|  | Incremental cost ($) | 331 | 49 | 230 | 219 | -25 | -49 | 157 |
|  | Incremental reduction (minutes) | 77 | 476 | 85 | 435 | 125 | 160 | 63 |
|  | ICER (95% CI) | **4.46** | **0.1** | **2.72** | **0.5** | **Dominant** | **Dominant** | **2.48** |
|  |  | **(1.06, 9.84)** | **(-0.32, 0.54)** | **(-0.10, 7.02)** | **(-0.04, 1.02)** | **(-2.5, 1.89)** | **(-2.95, 2.35)** | **(-21.53, 29.42)** |
|  | Note: dominant indicates reduction in cost and reduced time to be seen; dominated indicates greater incremental cost and increased time to be seen  Note: where there was a reduction in cost and a reduction in effectiveness (i.e. an increase in time) an ICER (incremental cost / incremental effectiveness) was not calculated  Note: all negative ICER values represent decreased cost and increased effectiveness  CI: credible interval; ICER: incremental cost effectiveness ratio; nc: not calculated | | | | | | | |
